# Let’s Learn About Emotions Program: Acceptability, Fidelity, and Students’ Mental Well-Being Outcomes for Finnish Primary School Children

**DOI:** 10.3390/children12091251

**Published:** 2025-09-17

**Authors:** Yuko Mori, Tiia Ståhlberg, Xiao Zhang, Kaisa Mishina, Sanna Herkama, Tarja Korpilahti-Leino, Terja Ristkari, Meeri Kanasuo, Saara Siirtola, Vesa Närhi, Hannu Savolainen, Susanna Hinkka-Yli-Salomäki, Shiho Torii, Kohei Matsubara, Kohei Kishida, Noriko Hida, Shin-ichi Ishikawa, Andre Sourander

**Affiliations:** 1 Department of Child Psychiatry, Research Centre for Child Psychiatry, University of Turku, 20014 Turku, Finland; tthuht@utu.fi (T.S.); xiao.zhang@utu.fi (X.Z.); kaisa.mishina@utu.fi (K.M.); sanna.herkama@utu.fi (S.H.); tarja.i.korpilahti-leino@utu.fi (T.K.-L.); terja.ristkari@utu.fi (T.R.); sushys@utu.fi (S.H.-Y.-S.);; 2 INVEST Research Flagship Center, University of Turku, 20014 Turku, Finland; 3 Department for Adolescent Psychiatry, Turku University Hospital, 20700 Turku, Finland; 4 Department of Teacher Education, University of Turku, 20500 Turku, Finland; 5 Department of Education, University of Jyväskylä, 40014 Jyväskylä, Finland; 6 School of Educational Sciences and Psychology, University of Eastern Finland, 70210 Kuopio, Finland; 7 Faculty of Human Studies, Seisen University, Hikone 521-1123, Japan; kmatsuba@mail.doshisha.ac.jp; 8 Graduate School of Human Development and Environment, Kobe University, Kobe 657-8501, Japan; kkishida@people.kobe-u.ac.jp; 9 Department of School education, Nara University of Education, Nara 630-8528, Japan; nhida@mail.doshisha.ac.jp; 10 Faculty of Psychology, Doshisha University, Kyotanabe 610-0394, Japan; ishinn@mail.doshisha.ac.jp; 11 Department of Child Psychiatry, Turku University Hospital, 20521 Turku, Finland

**Keywords:** mental health, well-being, school, intervention, universal, children, student, acceptability, fidelity, mixed-method

## Abstract

**Highlights:**

**Main findings?**

- The Let’s Learn About Emotions program was found to be highly acceptable to students, parents, teachers, and principals, and was delivered with high fidelity by classroom teachers.
- Parent reports indicated improvements in children’s conduct problems, hyperactivity, and peer problems, while student self-reports did not show similar benefits.

**Implication of the main finding?**

- The program is feasible, culturally appropriate, and suitable for implementation in Finnish schools, though refinements could further enhance its impact.
- Future research with a control design is needed to rigorously test effectiveness.

**Abstract:**

**Background/Objectives**: School-based universal mental health interventions offer an inclusive and scalable approach to promote mental health and well-being among children. This study evaluates the Let’s Learn About Emotions (Opitaan tunteista in Finnish) program, an evidence-based, teacher-led universal school-based intervention originally developed in Japan and culturally adapted for Finnish primary schools. **Methods**: A total of 512 fourth-grade students from 14 schools participated in the 12-week program during spring 2023. Using a mixed-methods design, we assessed (1) the program’s acceptability among students, parents, teachers, and school principals, (2) fidelity of implementation, and (3) changes in students’ mental well-being pre- to post-intervention. Quantitative data included standardized questionnaires with valid responses collected from 233 students at baseline and 209 students at post-intervention, as well as parents and teachers. Qualitative data were collected through focus group discussions involving parents, teachers, and school principals during spring 2024. **Results**: Acceptability was high across all respondent groups. Teachers adhered closely to the teaching manual, as confirmed by self-reports and direct classroom observations. Statistically significant improvements were observed in parent-reported conduct problems, hyperactivity, and peer problems, though student self-reports did not show similar benefits. **Conclusions**: The program was found to be both acceptable and culturally appropriate in the Finnish context. Findings from this study provide valuable insights for refining and improving the program for future implementation. To more rigorously examine its effectiveness, future studies should employ a randomized controlled trial design.

## 1. Introduction

School-based mental health interventions have expanded in response to growing recognition of the associations between emotional and behavioral problems and adverse outcomes such as severe psychopathology [1], school disengagement [2], and low educational attainment [3]. Mental health is an essential part of children’s overall health and it refers to a state of psychological and emotional well-being that enables them to cope with stress, function in daily life, and maintain healthy relationships [4]. Universal mental health interventions have particular appeal because they are designed to reach all children, can be integrated into children’s everyday environments, and do not exclude children who might potentially benefit from the intervention but not be selected for targeted interventions [5]. Evidence suggests that universal school-based interventions are cost effective [6] and can be successfully delivered by teachers with adequate training and support [7,8,9].

Common theoretical approaches in universal school-based mental health interventions include cognitive behavioral therapy (CBT), which targets unhelpful thoughts and behaviors [10]; mindfulness [11]; social and emotional learning (SEL), which develops skills such as emotional regulation, empathy, and relationship building [12]; and positive psychology (PPI), which focus on enhancing strengths, positive emotions, and resilience [13]. Research has shown the effectiveness of these interventions in improving various skills such as coping, help seeking, social, academic performance, and overall psychological well-being [14,15,16].

However, the evidence for these programs in preventing mental health problems has been inconsistent [10]. A large randomized controlled trial in the UK found that a universal school-based mindfulness program did not improve adolescent mental health [17], while some universal school-based mental health literacy programs have been linked to increased reported problems [18,19]. Universal programs may lead students to label normal experiences as problems, or that they may be too superficial to provide real therapeutic benefit, and potentially may even be harmful for some individuals [20,21]. Targeted interventions tend to yield more lasting and larger effects compared to universal interventions [22]. Specialized treatments are typically offered to children with moderate-to-severe or diagnosable problems. Importantly, evidence from the FRIENDS study indicates that a school-based mental health intervention for child anxiety achieved similar benefits to clinic-based delivery, suggesting that well-implemented school programs can provide outcomes comparable to specialist treatment in certain contexts [23]. 

There is limited data on the acceptability of these interventions among participants and facilitators [24,25] and on fidelity, defined as the extent to which an intervention is delivered as intended [26]. Examining acceptability, fidelity, efficacy, and sustainability is essential for refining interventions and ensuring their long-term effectiveness [27,28]. While mixed-methods studies are increasingly common in intervention research, there are still relatively few that apply this approach specifically to universal school-based mental health interventions [29]. Qualitative insights are crucial for understanding how interventions are experienced and implemented in real-world settings, including potential barriers and facilitators that are hard to capture via quantitative measures alone [30].

In Japan, a universal school-based intervention based on CBT, PPI, and SEL theories has been developed and successfully delivered to school settings (Universal Unified Prevention Program for Diverse Disorders, Up2-D2) [31]. Japanese studies showed that after receiving the Up2-D2 intervention, students demonstrated improvements in self-efficacy, social skills, and reductions in emotional and behavioral difficulties, while the program itself was also evaluated as highly acceptable and implemented with sufficient fidelity [31,32,33]. Furthermore, at-risk students with a total score above the normal level on emotional and behavioral difficulties (The Strengths and Difficulties Questionnaire > 18) prior to the intervention benefited the most from the universal intervention [33]. However, these findings are based on pilot studies using a single-group pre- and post-test design with small sample sizes, and no randomized controlled trials have been conducted. Previous studies of comparative interventions also showed that at-risk students scoring in the clinical range of anxiety [34] and emotional and behavioral difficulties [35] showed the strongest benefits from the interventions.

The Japanese Up2-D2 intervention was translated in Finnish and culturally adapted to fit the Finnish context [36]. The Finnish name for the intervention is Let’s Learn About Emotions (Opitaan tunteista in Finnish). This cross-cultural adaptation is particularly unique, as most school-based mental health interventions are typically developed in Western countries and later imported into non-Western contexts. In contrast, this intervention represents a rare example of an evidence-informed program originating in Asia and being adapted for use in a Western country. The aim of this study is to evaluate the acceptability, fidelity of the program, and changes in students’ mental well-being using a mixed-method design combining quantitative and qualitative methods. This approach was chosen to capture both measurable outcomes and in-depth perspectives from participants. Our research questions were (1) How do children, parents, and teachers perceive and accept the program? (2) To what extent do teachers implement the program as outlined in the teaching manual? (3) How does students’ mental well-being change from pre-intervention to post-intervention? The protocol of this study has been previously published [36].

## 2. Materials and Methods

### 2.1. Intervention

**Figure 1:**
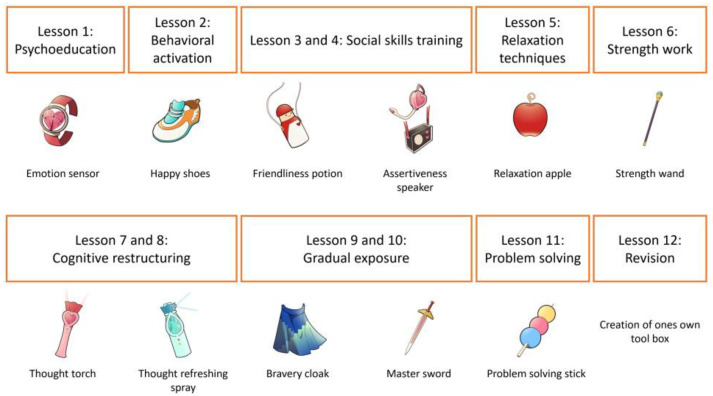
Content of the intervention and the tools representing the skills.

The Let’s Learn About Emotions program is a universal school-based intervention designed to promote mental well-being by enhancing socio-emotional skills in children aged 8 to 12. The program consists of 12 weekly lessons (each 45 min), using a story-like format with vivid illustrations. Three fictional children, each facing different challenges, such as anxiety, depressive thinking, or behavioral difficulties, meet a wise master who provides them with magical tools to manage their emotions. Each lesson introduces a new tool, and students practice using it through individual and group activities and homework assignments. Teachers play a key role in delivering the intervention. In this study, the teachers received a 2.5-h training workshop, a written teaching manual, and access to online support sessions. Each lesson was accompanied by an introductory video for teachers and printed materials, including a student book and A4-sized tool illustrations. The content of the intervention is illustrated in Figure 1 (see Figure S1 in the Supplementary Material for details of the three fictional children and the master). 

### 2.2. Participants

**Figure 2:**
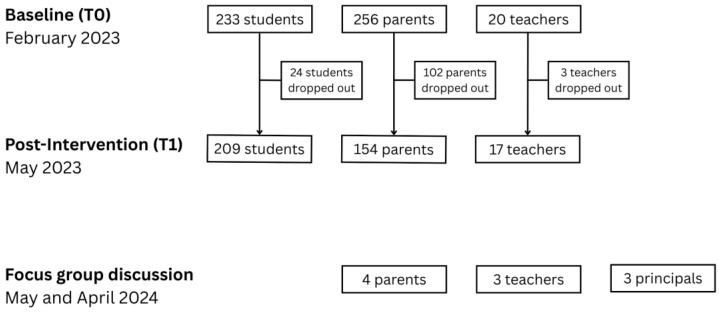
Study sample flowchart.

The quantitative data was based on students attending primary schools in the city of Hyvinkää in Southern Finland, and all fourth-grade students (aged 10–11) in the city were invited to participate in the program as a part of the national core curriculum. A total of 512 fourth-grade students from 36 classrooms across 14 public schools participated in the intervention during spring 2023. Inclusion criteria required that participants (1) had to be 4th-grade students enrolled in public schools in Hyvinkää city in spring 2023, (2) obtained active parental consent, and (3) provided their own assent to participate. Exclusion criteria included (1) students with severe intellectual disabilities, (2) students whose teachers were unable to conduct teaching in Finnish, and (3) students with very limited proficiency in the Finnish language. At baseline (T0, February 2023), we collected questionnaires from 233 students, 256 parents, and 20 teachers (Figure 2). Post-intervention (T1, May 2023), we received responses from 209 students (response rate: 89.7%), 154 parents (60.2%), and 17 teachers (85.0%). The qualitative data was based on three focus group discussions targeting different participant groups: parents, teachers, and school principals. Each focus group included 3–4 participants. In addition, due to scheduling issues, one teacher was interviewed individually. Although this differs from the nature of a focus group setting and lacks its dynamics, the interview was conducted and analyzed to enhance the richness of the data. The focus group discussions took place in March and April 2024, after classes had completed the program.

### 2.3. Procedure

Ethical approval for this research was obtained from the ethical board of the University of Turku. The study was also approved by the educational board of the City of Hyvinkää. For the quantitative evaluation, a single-group pre–post test design was used to assess changes over time. The measurement points were T0 before the intervention and T1 right after the completion of the intervention. Students and teachers completed the questionnaires on paper, whereas the parents filled the survey online. Participation was voluntary, and the anonymity and confidentiality of the participants were ensured. Active parental consent and child assent were obtained before participation. Teachers were provided with written information prior to completing the survey. The focus group discussions were held via Microsoft Teams and they lasted from 30 to 45 min. During the discussions, pre-constructed interview questions were asked in a structured manner and supplementary questions were presented when necessary. The discussions were recorded and the recordings were transcribed verbatim, resulting in 31 pages of text (Times New Roman, font size of 12, and a line spacing of 1.5). 

### 2.4. Measures

#### 2.4.1. Acceptability 

The acceptability of the intervention was assessed at T1 following the final session of the program, with data collected from students, teachers, and parents. A questionnaire was developed for this study based on the questionnaire used in the Japanese study [31] and the Client Satisfaction Questionnaire by Attkisson [37]. The student questionnaire comprised nine items, addressing aspects such as enjoyment of the sessions, clarity of the content, perceived utility of the program, and students’ intent to apply the program’s skills in real-life contexts. The internal consistency of the student acceptability scale was high (α = 0.862). For teachers, a total of 13 items evaluated the acceptability of the program, and an additional 10 items examined the acceptability of the preparatory workshop offered to educators prior to the program’s commencement. Internal consistency was strong for both teacher ratings of the program (α = 0.834) and the training (α = 0.830). Parents responded to five items addressing their satisfaction with the program’s information, the comprehensibility of both the homework and program content for their children, their overall evaluation of the program, and their likelihood of recommending it to others. Internal consistency for the parent acceptability scale was moderate (α = 0.609). For qualitative interviews, 6–8 pre-defined interview questions were used covering themes such as the perceived need for social–emotional skills training, participants’ views on the intervention and its use, and suggestions for its further development.

#### 2.4.2. Fidelity

Intervention fidelity was assessed with direct classroom observation and teacher self-reports. Two members of the study group passively observed 17 random lessons on-site, filling a fidelity checklist to assess the extent to which the intervention was delivered as intended according to the written teaching manual. The fidelity checklist was developed for this study based on the checklist used in the Japanese study [31], with slight modifications to fit the Finnish context. The checklist contained 7–13 questions for each lesson across four stages of program delivery: introduction, learning target skills, individual and group practices, and summary (e.g., practice measuring the intensity of emotions in groups.). In addition, teachers answered six fidelity questions following the final session of the program. The fidelity questions included three questions assessing the adherence to lesson content, time spent on lessons, and lesson coverage (e.g., Did you teach your class all lessons 1–12?).

#### 2.4.3. Early Efficacy Outcomes

The Strengths and Difficulties Questionnaire (SDQ) [38] was chosen to measure the early efficacy due to its extensive use in universal school-based interventions and well-established validity [39,40]. The SDQ consists of 25 items, divided into five subscales: emotional symptoms, conduct problems, hyperactivity, peer problems, and prosocial behavior, with each item scored from 0 to 2. The total score ranges from 0 to 40 (excluding the prosocial scale, which is scored inversely). The Finnish version has been translated and back-translated from English and has been validated across different age groups [41,42,43]. Among school-aged children, the SDQ total score has demonstrated a Cronbach’s alpha of 0.71 [43]. In the current study, internal consistency for the SDQ was acceptable across informants: for students, α = 0.754 at baseline (T0) and α = 0.743 at follow-up (T1); for parents, α = 0.740 at T0 and α = 0.765 at T1. Additionally, children’s perceived difficulties in emotions, concentration, behavior, and social interactions were assessed using the SDQ impact supplement. 

The other outcome measures included a shortened version of the Finnish Classroom Behavioral Climate (CBC) scale, which has demonstrated adequate reliability [44,45]. In the present study, the internal consistency of the CBC scale was lower for students (α = 0.447 at T0 and α = 0.407 at T1) but acceptable for teachers (α = 0.676 at T0 and α = 0.730 at T1). Both students and teachers provided CBC ratings. Loneliness was assessed through student and parent reports, while parents provided information on their child’s friendships, with questions adapted from the Children’s Depression Inventory scale [46,47]. Bullying victimization was assessed with three items on traditional (at school and outside school) and cyberbullying over the past six months (0 = Never to 3 = Almost every day). Perceived school environment was measured with seven items (e.g., feeling safe, teacher and peer support) rated on a 4-point scale (0 = Never to 3 = Always) [48]. Additionally, students’ self-reported knowledge of emotional awareness at T1 was assessed using a customized measurement developed by the Finnish research team. The measurement included 11 multiple-choice questions, each with three response options. The questions tested children’s learning and comprehension of the main points of the intervention (see Table S1 in the Supplementary Material for details of the measurement).

### 2.5. Data Analysis

The quantitative analyses were conducted using SPSS 28.0 [49]. For students’ background characteristics, frequencies and percentages were calculated for both T0 and T1. To assess the acceptability of the intervention, frequencies and percentages for each item on the student, teacher, and parent questionnaires were calculated. Observed fidelity scores were calculated as the percentage of completed checklist items out of the total possible items per lesson. The mean fidelity scores were calculated across all observed lessons. The comparison between T0 and T1 for students was examined using the paired *t*-test for all outcome variables, treating them as continuous variables. Paired *t*-tests were also conducted for parents’ and teachers’ reports: SDQ total scores and subscores, as well as children’s friendships and loneliness, were tested for parents, and the CBC was tested for teachers. The assumption of normality for all paired *t*-tests was assessed using the Shapiro–Wilk test, which indicated no significant deviation from normality (*p* = 0.155). The correlation between child and parent SDQ total scores was calculated using the Pearson correlation coefficient test. A two-sided *p*-value of less than 0.05 was considered significant. To evaluate students’ emotional awareness knowledge, the proportion of correct responses to each of the 11 multiple-choice questions was calculated. 

The qualitative data were analyzed with an abductive thematic analysis [50] to assess the acceptability of the program. Pre-defined themes reflected core dimensions of acceptability, namely usability and clarity; perceived usefulness; and contextual fit. First, the data were read several times and initial notes were taken. Second, new codes were generated systematically, reflecting the initial notes and relying on the pre-defined themes related to the core dimensions of acceptability. Third, codes were grouped into sub-themes that reflected both participants’ perspectives and existing conceptual understandings. Fourth, sub-themes and themes were reviewed in relation to the coded data and their coherence was ensured.

## 3. Results

At baseline (T0), 49.8% of students were male, 45.5% were female, and 3.0% identified as another gender (Table 1). At post-intervention (T1), the proportions were 51.7% male, 40.7% female, and 0.5% identifying as another gender. There was no significant difference in gender distribution between T0 and T1, χ^2^ (3, N = 436) = 7.72, *p* = 0.052. Most students spoke only Finnish as their mother tongue (90.1% at T0 and 85.6% at T1), with no significant change over time, χ^2^ (4, N = 429) = 2.01, *p* = 0.734. All teachers were female, with a mean age of 46.6 years (Standard deviation; SD = 11.2) at T0 and 47.4 years (SD = 12.1) at T1. Mothers were the most common respondents among parents (67.2% at T0 and 82.5% at T1).

### 3.1. Acceptability of the Program: Quantitative Results

Table 2 presents acceptability ratings of the program. The majority of students found the program easy to understand (69.3%) and felt they knew how to use its tools (61.2%). More than half of the students were satisfied with the program (55.6%), considered it good (54.1%), and liked the classes (50.0%). However, fewer students felt that the program made them feel better (27.7%) or helped them (26.6%). While 26.3% intended to use the tools outside school, only 19.0% had actually done so. The majority of parents were satisfied with the amount of information provided (86.6% responded “definitely true” or “somewhat true”). Most parents also rated the program positively, with 84.6% agreeing that it was good and 79.2% indicating they would recommend it to others. 

Teachers generally found the program and the training workshop acceptable and useful. All teachers (100%) agreed that the teacher’s material was easy to understand, and nearly all (94.1%) found the program’s structure easy to follow and that students could understand the material (93.8%). Support from schools varied, with 70.6% agreeing that their school supports the program’s content, but only 64.7% stating that their school actively supports its use. While 70.6% of teachers liked the program, 64.7% reported being satisfied with the program. All teachers (100%) agreed that the workshop provided a good background for the program and that the training was sufficient and of high quality. Most teachers felt that the training provided a good basis for teaching the program (88.2%), were satisfied with the content of the training materials (88.2%), found it easy to contact the research team (85.7%), were satisfied with the team’s responses (80%), and found the materials easy to use (75%). However, 42.9% felt that the introduction videos were not particularly useful and 44.4% did not find online meetings beneficial.

### 3.2. Acceptability of the Program: Qualitative Results

The qualitative analysis on the acceptability of the program identified three main themes: usability and clarity, perceived usefulness, and contextual fit, each capturing key aspects such as the program structure, content and visual design of the materials, the role of parents, the value of socio-emotional learning, improvement in everyday life, age appropriateness, and fit to the educational practices.

***Usability and clarity***

**Program structure, content, and visual design.** Principals emphasized that teaching emotional skills effectively is demanding and cannot be implemented without proper preparation (see Table 3). Both principals and teachers appreciated that the program was offered in book rather than digital format. The concrete book format was seen as user-friendly and convenient, with all necessary materials in one place. However, teachers noted some challenges: students can forget their books at home, and also purchasing books can be costly. Teachers also suggested that material could be more concise since the program consisted of a large amount of content, which sometimes exceeded the time available in lessons. Despite this, the overall structure of the program was considered well designed. Some parents declared that they actively discussed the content with their children, others said that they never saw the workbook or completed any assignments together. Regarding the program’s visual elements, all interviewed groups found them engaging and motivating for students, as this teacher explained: “*The advantage of this material is that it’s thoughtfully pre-designed, easy to implement, and visually appealing*”. 

**Role of parents.** Principals emphasized the value of parental involvement, and said that the program could be integrated into parent evenings, organizing class-specific information sessions and encouraging families to reinforce learned content at home. Parents also recognized their potential role as important and felt that their active involvement would benefit the program. They suggested that engagement could include family-oriented tasks and receiving summaries of the program’s structure, progress, and themes. Some parents also said that they could help their child to do the program’s homework together: “*To have materials that we could go through together, something to reflect on and discuss. That would have helped us engage with the topics more deeply*”. Some parents also reported that having more details would have supported meaningful conversations with their children and allowed them to offer better support. 

***Perceived usefulness***

**Need for improved socio-emotional skills.** Principals emphasized that emotional skills are a fundamental foundation for all learning and crucial for students’ future lives: “*I personally consider emotional skills to be important, if not the most important, because they form the foundation for everything else, we do in primary school. If these aspects aren’t in place in students’ minds, then all our efforts are in vain.”* They also evaluated that the program addresses current societal needs among children, such as increasing mental health problems, restlessness as well as emotional and behavioral difficulties. The need for the program was also reflected in principals’ observations that teachers were willing to implement it in their classrooms. Teachers echoed these same concerns.: “*Problems [in emotion and behavior] can be seen as restlessness, difficulty in concentrating and bullying. They also appear as fatigue, lack of motivation to work, or unwillingness to cooperate with either adults or peers.*” Parents found the program’s focus on emotional expression, particularly in the school context, to be valuable. They also reflected on how their child’s difficulties with emotional regulation could trigger challenging emotions in themselves, and expressed a desire for support in managing these situations in the future.

**Improvements in everyday life.** Principals pointed out that the program provided students essential tools to navigate peer relationships, such as conflict resolution and empathy, which often demand a significant amount of teachers’ time. One of the participating teachers highlighted that, especially towards the end of the program, students began sharing personal experiences, demonstrating their ability to transfer learned emotional skills into real-life situations. This participant also felt that discussions about emotions fostered greater trust between him/her and the students. Parents highlighted the importance of the program in helping children develop a deeper understanding of their emotional skills, improve their ability to verbalize emotions especially in a school environment, enhance their confidence in presenting in front of others, and prepare for a more independent life in general.

***Contextual fit***

**Suitability for the age group.** Principals evaluated the program fit to be fairly good for fourth graders as they are able to fluently read and write, are already familiar with the core concepts, and are able to operate with them, which all facilitate learning. However, among teachers, the material was considered partially more suitable for younger students: “*This feels more like early years material. Especially in the spring of 4th grade, you really start to see that pre-teen stuff coming out… So yeah, this material probably works better for younger kids.”* Parents, instead, considered the program as timely: they saw a clear need for an emotional skills program at this age, as children’s emotional lives begin to expand with the approach of adolescence and a wide range of emotions becomes increasingly visible both at home and at school. Parents also reported that their children were going through a developmental phase where emotional matters were becoming more significant and thought provoking and that children showed interest in the topics covered by the emotional skills program.

**Fit to educational practices.** Principals noted that the program aligns well with the national core curriculum and supports its implementation but not without problems: socio-emotional learning is not a subject itself and, therefore, finding a suitable time slot in the school timetable can be challenging. According to principals, teachers have the autonomy to decide when to implement the program, but this flexibility can also be a drawback, as it may lead to the program being overlooked. Therefore, they considered that a structured program encourages teachers to reserve dedicated time for this purpose. Teachers stressed that some dedicated time is needed: “*A program like this gives structure, and then it actually gets its own dedicated time. But that time has to come from somewhere else.*” Generally, they found it difficult at times to fit the program into the national core curriculum. Many of the participating teachers explained how they identified similar topics in relevant subjects and delivered the lessons as part of those subjects. Some of the participating teachers mentioned that social-emotional skills should be part of everyday practices - not only as a separately implemented program. One of the parents stated that [socio-emotional skills] “*absolutely should be part of the curriculum, because families come from such different starting points.*” The participant also pointed out that these skills were paid attention to in early childhood education but after that the good work somehow was “*cut off*”.

### 3.3. Fidelity

The fidelity scores were calculated as the percentage of completed checklist items per lesson. The mean fidelity score across 17 observed lessons was 82.3%, with individual lessons ranging from 63.2% to 100%. Overall, the fidelity of program implementation was high, with most lessons achieving over 80% adherence to the teaching manual. All teachers reported systematically going through the content of the lessons, with 47.1% indicating “always” and 52.9% “mostly”. Each lesson is designed to be taught in 45 min. While 41.2% spent exactly 45 min, 58.8% of teachers spent more than 45 min on average. The majority of teachers (88.2%) taught all 12 lessons, with 11.8% not completing the entire program.

### 3.4. Changes in Students’ Mental Well-Being

Table 4 presents the changes in students’ mental well-being. In the SDQ, based on self-reported results, there were significant increases in hyperactivity (*t*(205) = 2.39, *p* = 0.018) and decreases in prosocial behavior (*t*(205) = −2.51, *p* = 0.013) between T0 and T1. Conversely, the parent-reported SDQ scores indicated more positive changes, with improvements in conduct problems (*t*(146) = −2.31, *p* = 0.022), hyperactivity (*t*(146) = −3.33, *p* = 0.001), peer problems (*t*(146) = −2.11, *p* = 0.036), and perceived difficulties (*t*(146) = −3.86, *p* < 0.001). The agreement rate between child- and parent-reported SDQ total scores showed a medium effect, with Pearson correlation coefficients being significant for both T0 (r = 0.44, *p* < 0.001) and T1 (r = 0.38, *p* < 0.001) (See Supplementary Table S2). Additionally, students reported a worsening in the school environment (*t*(195) = −3.50, *p* < 0.001), whereas teachers did not report any significant changes in classroom behavior. No significant changes were observed in the other secondary measures between T0 and T1.

### 3.5. Emotional Awareness Knowledge

The percentage of students who correctly answered each question in the Emotional Awareness Knowledge assessment is presented in Figure S2. The results indicate notable variation in performance across questions. The highest percentage of correct responses was observed for lesson 6 (95.7%), followed by lesson 3 (85.2%) and lesson 9 (79.1%). In contrast, lesson 4 had the lowest accuracy (28.7%), suggesting a potential gap in understanding. Other questions exhibited moderate accuracy, ranging from 49.6% to 78.3%. 

**Table 4:** Changes in the outcomes in children, parents, and teachers.

	Outcome	Mean T0	Mean T1	Mean Difference (T1–T0)	Std. Deviation	Std. Error Mean	95% CI ^1^ of the Difference		*t*	df	Two-Sided *p*
							Lower	Upper			
Child	SDQ ^2^ total	9.40	9.92	0.524	4.225	0.294	−0.056	1.105	1.781	205	0.076
	SDQ emotional	3.00	3.10	0.107	2.069	0.144	−0.177	0.391	0.741	205	0.460
	SDQ conduct	1.68	1.87	0.189	1.478	0.103	−0.014	0.392	1.839	205	0.067
	SDQ hyperactivity	3.04	3.32	0.277	1.660	0.116	0.049	0.505	2.392	205	0.018
	SDQ peer relationships	1.68	1.63	−0.049	1.264	0.088	−0.222	0.125	−0.551	205	0.582
	SDQ prosocial	8.07	7.81	−0.257	1.471	0.102	−0.459	−0.055	−2.511	205	0.013
	SDQ perceived difficulties	0.61	0.43	−0.181	1.221	0.134	−0.447	0.086	−1.348	82	0.181
	Friendships	1.16	1.08	0.019	0.530	0.037	−0.053	0.092	0.524	206	0.601
	Loneliness	2.76	2.78	−0.073	0.857	0.060	−0.191	0.045	−1.222	204	0.223
	CBC ^3^ total	7.63	7.74	0.103	2.765	0.199	−0.288	0.495	0.519	193	0.604
	Bullying victimization	0.82	0.76	−0.059	1.157	0.081	−0.220	0.101	−0.729	201	0.467
	School environment	15.43	14.71	−0.724	2.899	0.207	−1.133	−0.316	−3.498	195	<0.001
Parent	SDQ total	8.65	7.67	0.082	1.620	0.134	−0.182	0.346	0.611	146	0.542
	SDQ emotional	1.84	1.46	−0.102	1.204	0.099	−0.298	0.094	−1.028	146	0.306
	SDQ conduct	1.65	1.46	−0.313	1.642	0.135	−0.581	−0.045	−2.311	146	0.022
	SDQ hyperactivity	3.34	3.03	−0.374	1.361	0.112	−0.596	−0.152	−3.333	146	0.001
	SDQ peer relationships	1.82	1.72	−0.190	1.094	0.090	−0.369	−0.012	−2.111	146	0.036
	SDQ prosocial	7.58	7.66	−0.017	1.613	0.210	−0.437	0.404	−0.081	58	0.936
	SDQ perceived difficulties	1.46	1.44	−0.980	3.078	0.254	−1.481	−0.478	−3.859	146	<0.001
	Child’s friendships	0.43	0.46	0.034	0.395	0.033	−0.030	0.098	1.043	146	0.299
	Child’s loneliness	0.07	0.09	0.014	0.261	0.022	−0.029	0.056	0.631	146	0.529
Teacher	CBC total	13.69	12.44	−1.250	3.152	0.788	−2.929	0.429	−1.586	15	0.133

^1^ CI = Confidence interval; ^2^ SDQ = The Strengths and Difficulties Questionnaire; ^3^ CBC = Classroom Behavioral Climate.

## 4. Discussion

This mixed-methods study demonstrates the acceptability, fidelity, and changes in mental well-being in the Let’s Learn About Emotions program among primary school children. Acceptability and fidelity were high, and the intervention seems feasible for teacher-led use in classroom settings in Finland. While parent-rated conduct, hyperactivity, and peer problems showed significant improvement, students themselves did not indicate similar benefits. 

The findings from both quantitative and qualitative data indicate that, overall, the program was evaluated as highly acceptable among students, parents, and teachers. Most students found the program easy to understand and reported knowing how to use its tools in everyday life. They were generally satisfied with the program; however, fewer students felt that the program significantly improved their well-being or helped them in a meaningful way. Notably, only 19.0% of students reported using the intervention techniques outside of school. One possible explanation for this is that students did not feel a strong need for using the learned skills, as reflected in their low baseline SDQ perceived difficulties score (0.61). Furthermore, since the acceptability assessment took place immediately after the intervention, students may not have had sufficient time to incorporate the skills into their daily lives. 

Consistent with student evaluations, parents also demonstrated a high level of acceptability across all aspects of the program. They expressed satisfaction with the amount of information provided, viewed the program positively, and indicated that they would recommend it to others. Qualitative interviews revealed that parents valued the program’s relevance and expressed interest in deeper involvement. Given that our program was designed to enhance children’s socio-emotional skills, which is a topic of growing concern among parents [51], this may have contributed to its favorable reception. Teachers similarly rated the program highly in all aspects of the program but identified areas for improvement in teacher training, particularly in the helpfulness of introductory videos and online meetings. Qualitative findings confirmed that while the concrete workbook format was appreciated for its usability and clarity, the extensive content sometimes exceeded available class time. Suggestions included condensing materials and ensuring alignment with existing classroom practices. These insights highlight specific aspects of the training that may need to be revised or enhanced to better support teachers in future implementations. 

The fidelity of program implementation was high, with a mean fidelity rate of 82.3% across all observed lessons, exceeding the 76.2% reported in a Japanese study [31]. Most lessons achieved over 80% fidelity to the teaching manual, and nearly all teachers systematically covered the lesson content. These findings align with prior research demonstrating that classroom teachers can effectively implement universal mental health interventions at school [7,8,9]. In Finland, where all teachers hold master’s degrees, their strong educational background may contribute to the high-quality delivery of interventions. Research has shown that facilitators who find an intervention acceptable are more likely to deliver it as intended [26]. The high levels of acceptability reported by teachers in our study may have contributed to the high fidelity observed in the program delivery. However, while each lesson was designed for 45 min, teachers often required more time to complete them. The program’s design can be further improved by implementing strategies to reduce the burden on teachers, such as simplifying the training materials and adjusting the amount of lesson content in each session.

This study found that parents perceived their children to benefit from the intervention, particularly in terms of behavioral problems. Previously, it has been shown that universal school-based programs are beneficial for behavioral outcomes [52,53]. On the other hand, behavioral symptoms are more easily detected by parents than emotional symptoms, which are most trustworthy when reported by children themselves [54,55]. In addition, hyperactivity and conduct problems, as assessed by the SDQ, are general markers of difficulties and could easily derive from internalizing problems such as depressive moods or anxiety. This overlap complicates the distinction between internalizing and externalizing disorders in self- and parent-report studies. The intervention incorporated multiple lessons on behavioral management, two lessons specifically on social skills, and emphasized classroom-based learning and group activities. These approaches may have reinforced both social skills and behavioral regulation, contributing to the observed benefits. On the other hand, parents could be biased to assess fewer problems in their children due to their expectations after the interventions. Parents were not involved much in the study design and, therefore, their participation in practicing and including the skills in the child’s daily life was limited.

Students themselves reported no significant improvement following the intervention. Instead, they reported a significant increase in hyperactivity, a decrease in prosocial behavior, and a deterioration in the school environment. One hypothesis for our findings is that improved mental health awareness might actually lead to prevalence inflation, which means that people become too self-aware and start to overinterpret normative emotions and conditions as problems and disorders [20]. This could even worsen the mental health in some individuals, and the potential harms of universal interventions should be considered carefully [21]. However, universal programs equip all students with emotional awareness and coping skills that may enhance resilience and prevent the onset of mental health problems [55]. Given the rising incidence of internalizing symptoms, particularly among girls [56,57], interventions that enhance mental health and resilience while equipping students with strategies to manage anxiety are increasingly justified.

**Strengths and limitations**

The results of this study should be interpreted with several limitations in mind. First, the lack of a control condition prevents attributing observed changes solely to the intervention. Other factors, such as unmeasured environmental influences, concurrent life events, and normal child development, may have contributed to the outcomes. A control group was not included, as the primary aim of this initial feasibility study was to evaluate the program’s acceptability, implementation fidelity, and suitability in the Finnish school context, rather than to establish causal effects. Rather than assessing the program’s effectiveness, this study aimed to explore the changes in students’ mental well-being using a pre–post design. The findings serve as a foundation for future large-scale randomized studies. Another limitation is the low response rates, particularly among parents. The study design does not allow conclusions about those who chose not to participate or did not complete the post-intervention assessment. In addition, the participants were not asked about the potential adverse effects of the intervention. 

Regarding the qualitative data, due to scheduling issues, one teacher was inter-viewed individually. This inevitably influenced the nature of the collected data, since the group interaction and dynamics were absent, representing a potential limitation. However, the data was included in the analysis to increase the richness of the description. To ensure trustworthiness the following actions were taken. First, multiple data sources were included in the study design and participants were purposefully selected to represent diverse experiences supporting transferability. Second, a thick description of the context and participants was emphasized. However, focus groups were conducted in Finnish school settings in one city, which may affect transferability. Third, researchers held regular meetings and used multiple coders to enhance trustworthiness. Researchers reflected on their own professional backgrounds, acknowledging potential influences on interpretations. Notably, the identified themes align with similar studies in school contexts, suggesting broader relevance.

Despite these limitations, the study also has notable strengths. One key strength is the use of multiple informants who provided a comprehensive perspective on the intervention’s impact. The combination of quantitative and qualitative methods added value, and feedback from principals, teachers and parents about the acceptability of program provided important input for further development. Additionally, the structured implementation of the teaching manual and provision of the teacher training workshop enhanced the consistency and fidelity of program delivery. 

## 5. Conclusions

This study identified that the Let’s Learn About Emotions program is acceptable in Finnish primary school settings but the benefits of the intervention require future research. Areas for improvement include enhancing the program’s relevance for students, condensing lesson content to fit classroom schedules, and creating opportunities for stronger parental involvement. Future research should also focus on identifying appropriate instruments (such as measures of self-efficacy) that may better capture the program’s intended effects. In addition, children’s ratings of acceptability should be assessed more thoroughly, for example, through interviews or by employing more comprehensive measurement tools. Finally, larger randomized controlled trials are needed to confirm effectiveness and rule out confounding factors such as regression to the mean. The study underscores the importance of piloting interventions before wider implementation, allowing for necessary refinements to enhance their implementation and impact. Furthermore, these results provide a foundation for expanding the intervention to a larger number of schools and conducting a randomized controlled trial to assess its effectiveness more rigorously. 

## Figures and Tables

**Table 1:** Study sample characteristics at baseline and post-intervention.

Characteristic	Categories	Baseline (T0)	Post-Intervention (T1)
Students		(*n* = 233)	(*n* = 209)
Gender	Male	116 (49.8%)	108 (51.7%)
	Female	106 (45.5%)	85 (40.7%)
	Other	7 (3.0%)	1 (0.5%)
	Do not want to tell	4 (1.7%)	10 (4.8%)
	Missing	0 (0%)	5 (2.4%)
Mother tongue	Finnish only	210 (90.1%)	179 (85.6%)
	Swedish only	3 (1.3%)	6 (2.9%)
	Finnish and Swedish	7 (3.0%)	3 (1.4%)
	Finnish and other	2 (0.9%)	3 (1.4%)
	Others	10 (4.3%)	8 (3.8%)
	Missing	1 (0.4%)	10 (4.8%)
Teachers		(*n* = 20)	(*n* = 17)
Gender	Male	0 (0%)	0 (0%)
	Female	20 (100%)	20 (100%)
	Other	0 (0%)	0 (0%)
	Do not want to tell	0 (0%)	0 (0%)
	Missing	0 (0%)	0 (0%)
Age (Mean ± SD ^1^)	-	46.6 (11.2)	47.4 (12.1)
Parents		(*n* = 256)	(*n* = 154)
Relationship to student	Mother	172 (67.2%)	127 (82.5%)
	Father	31 (12.1%)	17 (11.0%)
	Both parents	40 (15.6%)	7 (4.5%)
	Other	4 (1.6%)	2 (1.3%)
	Missing	9 (3.5%)	1 (0.7%)

^1^ SD = Standard deviation.

**Table 2:** Acceptability of program among students, parents, and teachers (quantitative results).

Questions	*n* (%)	*n* (%)	*n* (%)	*n* (%)
Students (N = 209)	Agree	Neither agree nor disagree	Disagree	
Content was easy to understand	142 (69.3)	52 (25.4)	11 (5.4)	
I understood how to use tools	126 (61.2)	69 (33.5)	11 (5.3)	
I was satisfied with the program	115 (55.6)	78 (37.7)	14 (6.8)	
Program was good	112 (54.1)	82 (39.6)	13 (6.3)	
I liked the classes	103 (50.0)	84 (40.8)	19 (9.2)	
Program made me feel better	57 (27.7)	109 (52.9)	40 (19.4)	
Program helped me	55 (26.6)	118 (57.0)	34 (16.4)	
I will use tools in or outside of school	54 (26.3)	82 (40.0)	69 (33.7)	
I have used tools in or outside of school	39 (19.0)	77 (37.6)	89 (43.4)	
Parents (*n* = 154)	Definitely true	Somewhat true	Not true at all	Cannot say
I was satisfied with the amount of information received about the program	72 (48.3)	57 (38.3)	6 (4.0)	14 (9.4)
Program seemed good	94 (63.1)	32 (21.5)	0 (0)	23 (15.4)
I would recommend the program for others	91 (61.1)	27 (18.1)	1 (0.7)	30 (20.1)
Homework was easy to understand for the child	68 (45.6)	43 (28.9)	1 (0.7)	37 (24.8)
Content of the program was easy to understand for the child	70 (47.0)	40 (26.8)	2 (1.3)	37 (24.8)
Teachers (*n* = 17)	Agree	Neither agree nor disagree	Disagree	
Program				
Teacher’s material was easy to understand	17 (100)	0 (0)	0 (0)	
It was easy to follow structure while teaching	16 (94.1)	0 (0)	1 (5.9)	
Students were able to easily understand the material	15 (93.8)	1 (6.3)	0 (0)	
Students liked the program	15 (88.2)	2 (11.8)	0 (0)	
I got help from the program	15 (88.2)	2 (11.8)	0 (0)	
I have made use of program’s content in my work	15 (88.2)	1 (5.9)	1 (5.9)	
I will make use of program’s content in my work	15 (88.2)	2 (11.8)	0 (0)	
Learning the teaching material took feasible amount of time	14 (82.4)	2 (11.8)	1 (5.9)	
I would recommend the program to others	13 (76.5)	2 (11.8)	2 (11.8)	
My school supports program’s content	12 (70.6)	5 (29.4)	0 (0)	
I liked the program	12 (70.6)	4 (23.5)	1 (5.9)	
My school supports using the program	11 (64.7)	6 (35.3)	0 (0)	
I was satisfied with the program	11 (64.7)	4 (23.5)	2 (11.8)	
Training workshop				
Training gave good background for the program	17 (100)	0 (0)	0 (0)	
I was satisfied with the amount of training	14 (100.0)	0 (0)	0 (0)	
I was satisfied with the quality of training	5 (100.0)	0 (0)	0 (0)	
Training gave good basis for teaching the program	15 (88.2)	0 (0)	2 (11.8)	
Written teacher’s material was comprehensive	15 (88.2)	2 (11.8)	0 (0)	
It was easy to contact the research team	12 (85.7)	2 (14.3)	0 (0)	
Research team gave satisfactory answers	12 (80.0)	2 (13.3)	1 (6.6)	
Written teacher’s material was easy to use	12 (75.0)	4 (25.0)	0 (0)	
Introduction videos were useful	5 (35.7)	3 (21.4)	6 (42.9)	
Online meetings during the course aided my work	0 (0)	5 (55.6)	4 (44.4)	

**Table 3:** Acceptability of program described by the principals, teachers, and parents (qualitative results).

Themes	Sub-themes
Usability and clarity	Program structure, content, and visual design Role of parents
Perceived usefulness	Need for improved socio-emotional skills Improvements in everyday life
Contextual fit	Suitability for the age group Fit to educational practices

## Data Availability

The data presented in this study are available on request from the corresponding author due to privacy and ethical reasons.

## Supplementary Materials

The following supporting information can be downloaded at: https://www.mdpi.com/article/10.3390/children12091251/s1, Figure S1: Finnish book page of the Let’s Learn About Emotions program featuring three fictional child characters and the master of emotions; Figure S2: Percentage of students answering correctly to emotional awareness knowledge questions (N = 115); Table S1: The questions used to measure students’ emotional awareness knowledge (assessed at T1); Table S2: Correlation between the total SDQ score for both students and parents, in T0 and T1.

## Abbreviations

The following abbreviations are used in this manuscript:

CBT	Cognitive Behavioral Therapy
SEL	Social and Emotional Learning
PPI	Positive Psychology
Up2-D2	Universal Unified Prevention Program for Diverse Disorders
SDQ	The Strengths and Difficulties Questionnaire
CBC	Classroom Behavioral Climate
