# Supplementary material for: Let’s Learn About Emotions Program: Acceptability, Fidelity, and Students’ Mental Well-Being Outcomes for Finnish Primary School Children
Source: Children (Basel). 2025 Sep 17;12(9):1251. doi: 10.3390/children12091251 (PMC12468062; doi:10.3390/children12091251)
Supplement: Supplementary file 1 [file children-12-01251-s001.zip › children-3819559-supplementary.pdf]

Supplementary files

Table S1. The questions used to measure students' emotional awareness knowledge (assessed at T1).

1.	The emotion sensor helps to recognize<br>A) Uncomfortable emotions and their intensities<br>B) All emotions and their intensities<br>C) Pleasant emotions and their intensities
2.	When feeling down and nothing seems interesting, you should<br>A) Rest<br>B) Be alone<br>C) Do enjoyable things
3.	A sip of Friendly Brew helps you<br>A) Not to care about feeling bad<br>B) Speak kindly to friends<br>C) Be more diligent
4.	It is important to know how to ask and decline firmly, because<br>A) Otherwise, you might not get what you want<br>B) Otherwise, you might end up doing things you don't want to do<br>C) Both of the above
5.	Relaxation exercises<br>A) Help relax the body and mind<br>B) Help calm an unpleasant emotional state<br>C) Both of the above
6.	Strengths<br>A) Kindness can be a strength<br>B) Only extremely talented students have strengths<br>C) Recognizing one's strengths is not useful
7.	The thought torch<br>A) Helps to recognize one's thoughts<br>B) Helps in the dark<br>C) Changes your negative thoughts into more positive ones
8.	When you spray Thought Freshener<br>A) You spray kindness<br>B) You relax<br>C) You change negative thoughts into more positive ones
9.	Facing a challenge or fear is worth it because<br>A) The uncomfortable feeling related to the situation will ease over time, and you can overcome the challenge<br>B) You get to use the Courage Cloak, which makes you invisible<br>C) Both of the above
10.	Challenges are broken down because<br>A) We want to get rid of the challenge as quickly as possible<br>B) Facing the challenge is easier step by step<br>C) The challenge doesn't fit under the Courage Cloak otherwise
11.	With the Solution Baton<br>A) Challenges are broken down in three steps<br>B) Puzzles are solved in three steps<br>C) Problems are solved in three steps

Table S2. Correlation between the total SDQ score for both students and parents, in T0 and T1.

	**SDQ total score student (T0)**	**SDQ total score student (T1)**	**SDQ total score parents (T0)**	**SDQ total score parents (T1)**
SDQ total score student (T0)	-			
SDQ total score student (T1)	0.713<br>< 0.001	-		
SDQ total score parents (T0)	0.439<br>< 0.001	0.387<br>< 0.001	-	
SDQ total score parents (T1)	0.489<br>< 0.001	0.377<br>< 0.001	0.856[**]<br>< 0.001	-

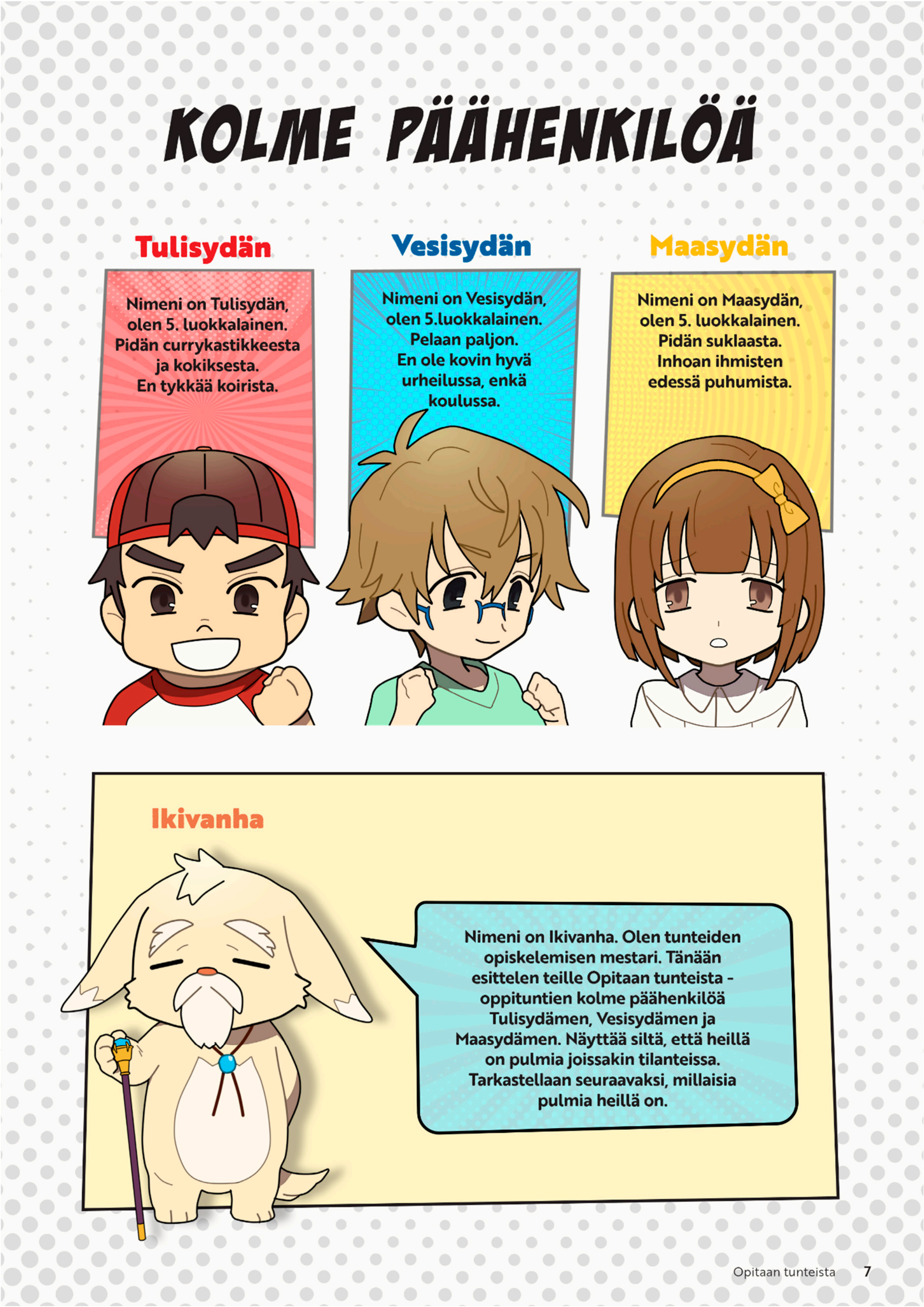

Figure S1. Finnish book page of the Let's Learn About Emotions program featuring three fictional child characters and the master of emotions.

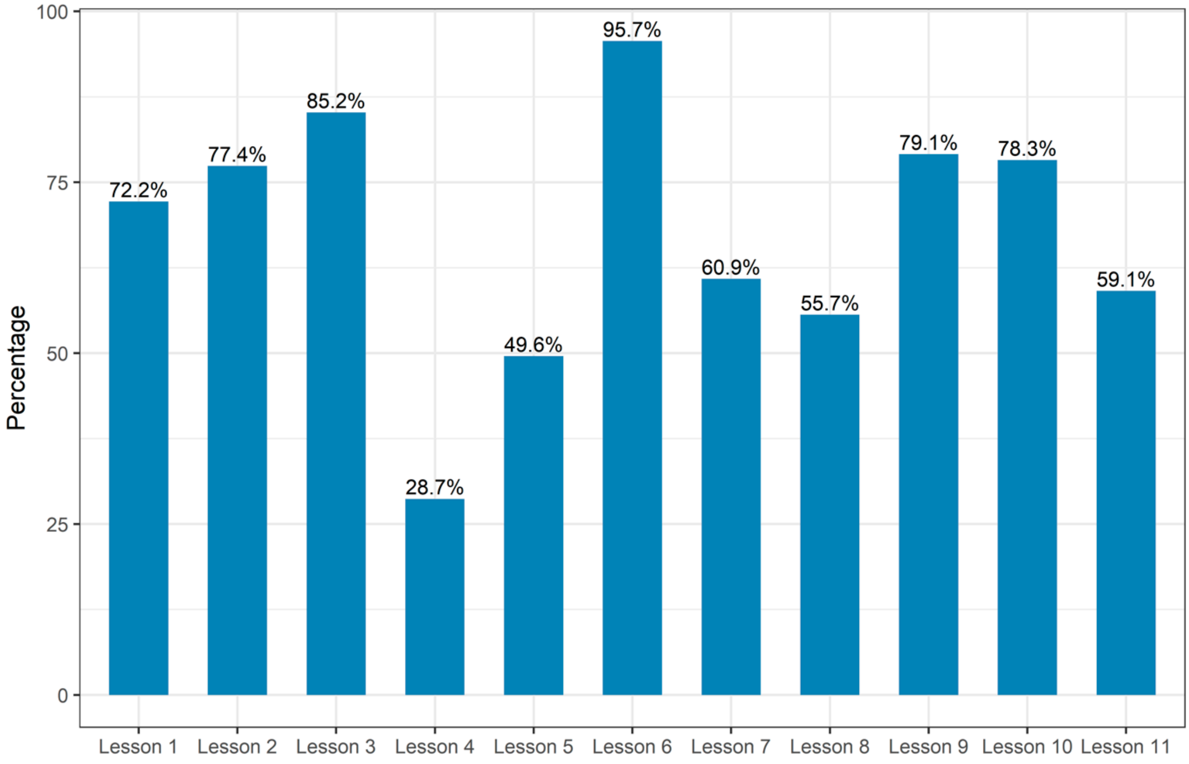

Figure S2. Percentage of students answering correctly to emotional awareness knowledge questions (N = 115).
